# Gender Differences in Faculty Perceptions of Mentorship and Sponsorship

**DOI:** 10.1001/jamanetworkopen.2023.55663

**Published:** 2024-02-12

**Authors:** Christina M. Cutter, Kent A. Griffith, Isis H. Settles, Abigail J. Stewart, Eve A. Kerr, Eva L. Feldman, Reshma Jagsi

**Affiliations:** 1 Department of Emergency Medicine, University of Michigan, Ann Arbor; 2 Center for Bioethics and Social Sciences in Medicine, University of Michigan, Ann Arbor; 3 Department of Biostatistics, University of Michigan, Ann Arbor; 4 Department of Psychology, University of Michigan, Ann Arbor; 5 Department of Women’s and Gender Studies, University of Michigan, Ann Arbor; 6 Department of Internal Medicine, University of Michigan, Ann Arbor; 7 VA Center for Clinical Management Research, Department of Veterans Affairs, Ann Arbor, Michigan; 8 Department of Neurology, University of Michigan, Ann Arbor; 9 Department of Radiation Oncology, Emory University, Atlanta, Georgia

## Abstract

This survey study examines gender differences in mid- to senior-career faculty experiences of receiving and providing mentorship and sponsorship during early career development.

## Introduction

Despite increasing awareness of the importance of mentorship and sponsorship in academic medicine and gender differences in their receipt during early career stages, knowledge is lacking regarding the experiences and perceptions of senior faculty.^1,2,3^ We surveyed a national cohort of mid- to senior-career faculty about their experiences with receiving and providing mentorship and sponsorship and their perceptions regarding the costs and benefits of these relationships.

## Methods

For this survey study,^4^ between August 1, 2021, and April 30, 2022, we mailed and/or emailed first-time recipients of National Institutes of Health K08 or K23 career development awards from 2006 to 2009 a 12-page questionnaire, cash incentive, and cover letter outlining elements necessary for informed consent, with a waiver of documentation by the University of Michigan institutional review board, which approved this study. A multistep approach to survey administration was used, with a modified Dillman approach for nonrespondents.^5^ This study follows the AAPOR best practices for survey research.

This cohort received early-career structured mentoring and are now positioned to reflect on their experiences in receiving and providing mentorship and sponsorship. Participants were asked about their experiences in receiving and providing various forms of mentorship and sponsorship and their perceptions surrounding the costs and benefits of mentoring, measured using an adapted established instrument.^6^ Self-reported gender was the primary independent variable of interest. Bivariate and adjusted analyses from multivariable models, including self-reported race (grouped by investigators as Asian, underrepresented in medicine [American Indian or Alaska Native, Black or African American, Hispanic or Latino, Native Hawaiian or Pacific Islander, or participant write-in], or White), academic rank, K-award year and type, and specialty, were performed using SAS, version 9.4 (SAS Institute Inc). A 2-sided *P* < .05 was considered significant.

## Results

Of 1430 individuals surveyed, 915 responded (64.0%); respondents did not differ significantly from nonrespondents by gender.^4^ The analytic sample was restricted to respondents remaining in academia (830 [90.7%]) and, for gender comparisons, to those identifying as men (422 [50.8%]) or women (385 [46.4%]). Most respondents identified as White (572 [68.9%], compared with 169 [20.4%] Asian, 66 [7.9%] underrepresented in medicine, and 23 [2.8%] with no response). Respondents’ academic ranks were predominantly associate (337 [40.6%]) or full (459 [55.3%]) professor.

There was a gender difference in mentorship structure, with 73 women (19.0%) responding they had “very much” turned to colleagues for peer mentorship vs 43 men (10.2%; *P* = .008). After adjustment (Figure), women were less likely to report sponsorship surrounding receiving or providing invitations to write editorials and receiving invitations to serve as visiting professors. Gender differences in the perceived benefits and costs of mentoring were also apparent (Table). Men were more likely to endorse benefits of organizational recognition, protégés being loyal supporters, and reliving their lives through their protégés and more likely to cite costs consequent to perceived risk in mentoring someone of a different sex. The highest item mean scores were mentoring being a positive career experience and time commitment as a drawback.

**Figure.**
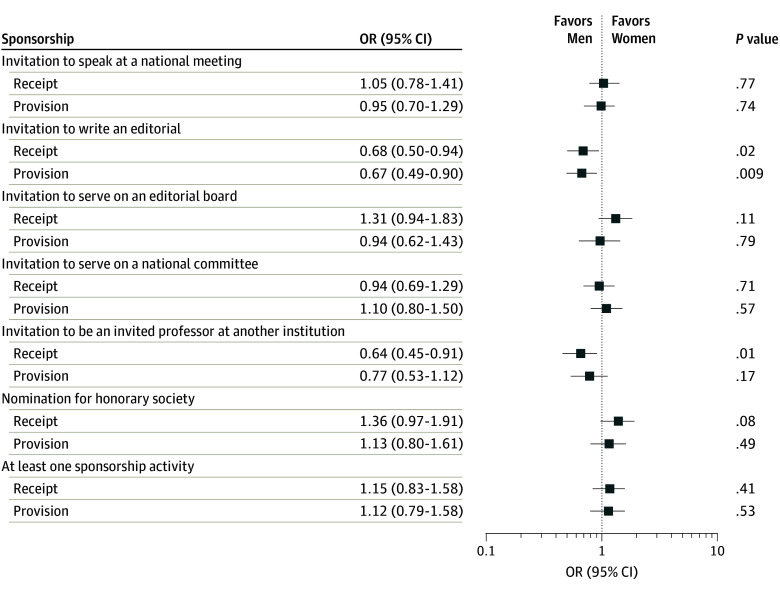
Forest Plot Illustrating Gender Differences in Self-Reported Sponsorship Opportunities Participants were provided the following definition: “Sponsorship is when somebody in a position of power or authority leverages their position of influence or privilege in a way that benefits a protégé.” They were then asked to check all that apply to the following question stems: “Thinking about all of your mentors and other senior colleagues, have any of them sponsored you in the following ways in the past 2 years?” and “Thinking about all of your protégés, have you helped to sponsor them in the following ways in the past 2 years?” Odds ratios (ORs) were adjusted for the independent effects of respondent self-reported race (grouped by investigators as Asian, underrepresented in medicine, or White), academic rank (assistant professor/other, associate professor, or full professor), K-award type (K08 or K23), year of K-award (2006, 2007, 2008, or 2009), and specialty (basic sciences or non-MD; clinical specialties for women, children, and families; hospital-based specialties; medical specialties; or surgical specialties). Gender was self-reported, as detailed previously^4^; 2 respondents who identified as nonbinary and 21 respondents who did not respond were excluded from these analyses.

**Table.** Perceived Costs and Benefits of Mentoring Others by Gender Identity^a^

Item	Mean score (95% CI)			*P* value^c^	Adjusted *P* value^d^
	Total (n = 830)^b^	Women (n = 385)	Men (n = 422)		
**Mentorship benefit scale**					
Summary score	4.97 (4.91-5.03)	4.85 (4.77-4.94)	5.09 (5.02-5.17)	<.001	<.001
1. Serving as a mentor can be one of the most positive experiences of one’s career.	6.30 (6.25-6.36)	6.33 (6.25-6.42)	6.30 (6.22-6.37)	.54	.37
2. One’s job performance is likely to improve when one becomes a mentor.	5.53 (5.45-5.62)	5.47 (5.35-5.60)	5.60 (5.49-5.71)	.14	.31
3. Mentors can count on their protégés to be loyal supporters.	4.74 (4.65-4.82)	4.57 (4.44-4.71)	4.89 (4.79-5.00)	<.001	.003
4. Mentors obtain positive recognition in their organization for assuming a mentoring role.	4.88 (4.79-4.97)	4.76 (4.62-4.90)	5.05 (4.93-5.17)	.002	.005
5. Mentors are able to relive their lives through their protégés.	3.39 (3.29-3.49)	3.14 (2.99-3.29)	3.63 (3.49-3.77)	<.001	<.001
**Mentorship cost scale**					
Summary score	3.10 (3.05-3.15)	3.00 (2.92-3.07)	3.19 (3.12-3.26)	<.001	.001
6. Being a mentor is more trouble than it’s worth.	2.36 (2.27-2.44)	2.38 (2.25-2.51)	2.31 (2.20-2.43)	.45	.58
7. Mentors run the risk of being displaced by successful protégés.	2.29 (2.20-2.38)	2.19 (2.06-2.32)	2.36 (2.24-2.49)	.06	.08
8. Members of the organization often view mentors as playing favorites with their protégés.	3.48 (3.38-3.58)	3.33 (3.18-3.48)	3.58 (3.45-3.72)	.02	.07
9. Choosing a poor protégé is a negative reflection on the mentor’s judgment.	3.26 (3.17-3.36)	3.16 (3.03-3.30)	3.36 (3.22-3.49)	.05	.07
10. The major drawback of being a mentor is the time commitment.	4.71 (4.61-4.81)	4.77 (4.62-4.92)	4.67 (4.53-4.81)	.35	.82
11. Mentoring someone of a different sex from one’s own is risky.	2.23 (2.15-2.32)	2.02 (1.90-2.13)	2.40 (2.26-2.53)	<.001	<.001

^a^ The above-modified mentorship scale is an adaptation of the scale developed by Ragins and Scandura.^6^ The original scale measured mentorship benefits and costs with 5 subscales, each with several questions, for each of the 2 constructs (benefits and costs). Our adaptation for this survey selected only 1 question for each subscale. Therefore, we asked participants 10 questions (5 representing benefits and 5 representing costs) with terminology reflecting the original instrument.^6^ We also asked a new question for cost: “Mentoring someone of a different sex from one’s own is risky.” Exploratory factor analysis suggests that a 2-factor solution explains a majority of the total variance and that the 5 benefit questions have positive factor loadings on the first factor and the 6 cost questions have positive factor loadings on the second factor, suggesting 2 factors that can be named mentorship benefit and mentorship cost, as expected. Benefit and cost summary scores were calculated as the mean of the valued questions, with higher values indicating greater benefit or cost. Question responses were on a 7-point Likert scale ranging from strongly disagree to strongly agree. The questions comprising the benefit scale were all significantly positively correlated, with an overall standardized Cronbach α = 0.68, suggesting moderate agreement. The questions comprising the cost scale and our newly created question were also all significantly positively correlated, with an overall standardized Cronbach α = 0.65, again suggesting moderate agreement.

^b^ Total (n = 830) comprises respondents identifying as women (n = 385), men (n = 422), nonbinary (n = 2), and those who did not answer (n = 21).

^c^ Reported *P* values represent the unadjusted comparison between respondents identifying as women and men, excluding those identifying as nonbinary (n = 2) and who did not answer (n = 21).

^d^ Reported *P* values represent the comparison between respondents identifying as women and men when adjusting for respondent self-reported race (grouped by investigators as Asian, underrepresented in medicine, or White), academic rank (assistant professor/other, associate professor, or full professor), K-award type (K08 or K23), year of K-award (2006, 2007, 2008, or 2009), and specialty (basic sciences or non-MD; clinical specialties for women, children, and families; hospital-based specialties; medical specialties; or surgical specialties).

## Discussion

The study findings illuminate gender differences in perceived costs and benefits of developmental relationships and suggest that gender differences in mentorship and sponsorship at early career phases may persist well into the careers of established faculty.^2,6^ Women reported fewer instances of sponsorship for high-profile public opportunities, and men endorsed greater concern surrounding mentoring someone of a different sex. Study limitations include the select cohort focus, which may not reflect faculty experiences more broadly and limits generalizability, especially to those who did not have early-career structured mentoring. Similarly, exclusion of respondents who left academia, whose experiences may have differed, is a limitation that merits attention. Overall, our study advocates for better institutional support for developmental relationships in academic medicine, including compensating for the time commitment, and highlights a need to address men’s concerns surrounding mentoring women, by both encouraging it and providing clear guidelines, with critical implications for workforce vitality and equity.^3^

## Notes

Supplement.
Data Sharing Statement
