# Supplementary material for: Gender Differences in Faculty Perceptions of Mentorship and Sponsorship
Source: JAMA Netw Open. 2024 Feb 12;7(2):e2355663. doi: 10.1001/jamanetworkopen.2023.55663 (PMC10862153; doi:10.1001/jamanetworkopen.2023.55663)
Supplement: Supplement. — Data Sharing Statement [file jamanetwopen-e2355663-s001.pdf]

Data Sharing Statement

Cutter. Gender Differences in Faculty Perceptions of Mentorship and Sponsorship. *JAMA Netw Open*. Published February 12, 2024. doi:10.1001/jamanetworkopen.2023.55663

Data
Data available: No

Additional Information
Explanation for why data not available: Data will not be shared to protect participant privacy.
